# Anti-inflammatory effects of diet and caloric restriction in metabolic syndrome

**DOI:** 10.1007/s40618-021-01547-y

**Published:** 2021-03-08

**Authors:** L. Montefusco, F. D’Addio, C. Loretelli, M. Ben Nasr, M. Garziano, A. Rossi, I. Pastore, L. Plebani, M. E. Lunati, A. M. Bolla, M. D. Porta, G. Piuri, F. Rocchio, A. Abdelsalam, E. Assi, M. Barichella, A. Maestroni, V. Usuelli, L. Loreggian, F. Muzio, G. V. Zuccotti, R. Cazzola, P. Fiorina

**Affiliations:** 1 Division of Endocrinology, ASST Fatebenefratelli-Sacco, Milan, Italy; 2 International Center for T1D, Pediatric Clinical Research Center Romeo Ed Enrica Invernizzi, DIBIC L. Sacco, Università Di Milano, Milan, Italy; 3 Nephrology Division, Boston Children’s Hospital, Harvard Medical School, 300 Longwood Ave, Boston, MA 02115 USA; 4 Department of Biomedical and Clinical Sciences, “L. Sacco”, Università Di Milano, Milan, Italy; 5 Department of Biochemistry and Biotechnology, Heliopolis University, Cairo, Egypt; 6 Clinical Nutrition Unit, Parkinson Institute, ASST Gaetano Pini-CTO, Milan, Italy; 7 Clinical Nutrition and Dietetic Unit, Luigi Sacco Hospital, ASST Fatebenefratelli Sacco, Milan, Italy; 8 Department of Pediatrics, V. Buzzi Childrens’ Hospital and Pediatric Clinical Research Center Romeo Ed Enrica Invernizzi, DIBIC L. Sacco, Università Degli Studi Di Milano, Milan, Italy

**Keywords:** Metabolic syndrome, Caloric restriction, Pro-inflammatory cytokines, Cholesteryl ester transfer protein, Lipids

## Abstract

**Background:**

Weight loss in patients with metabolic syndrome has positive effects on cardiovascular and type 2 diabetes risks, but its effects on peripheral cytokines and lipid profiles in patients are still unclear.

**Aim:**

To determine the effects of diet-induced weight loss on metabolic parameters, lipids and cytokine profiles.

**Methods:**

Eighteen adult males with metabolic syndrome (defined according to IDF 2009) and Body Mass Index (BMI) between 25 and 35 kg/m^2^ were subjected to a balanced hypocaloric diet for 6 months to reach at least a 5% body weight loss.

**Results:**

After weight loss, a significant improvement in BMI, waist circumference, insulin, fasting blood glucose and HOMA-IR (homeostasis model assessment of insulin resistance) was observed. The analysis of LDL (low-density lipoprotein cholesterol) and HDL (high-density lipoprotein cholesterol) lipoproteins showed a change in their composition with a massive transfer of triacylglycerols from HDL to LDL. This was associated with a significant reduction in peripheral pro-inflammatory cytokines such as IL-6, TNF-α, IL-8 and MIP-1β, leading to an overall decreased inflammatory score. An interesting positive correlation was also observed among peripheral cytokines levels after diet and peripheral levels of CETP (cholesteryl ester transfer protein), an enzyme with a key role in lipid change.

**Conclusion:**

Weight loss through caloric restriction is associated with an improvement in peripheral lipid and cytokine profiles that may play a major role in improving cardiovascular risk.

**Graphic abstract:**

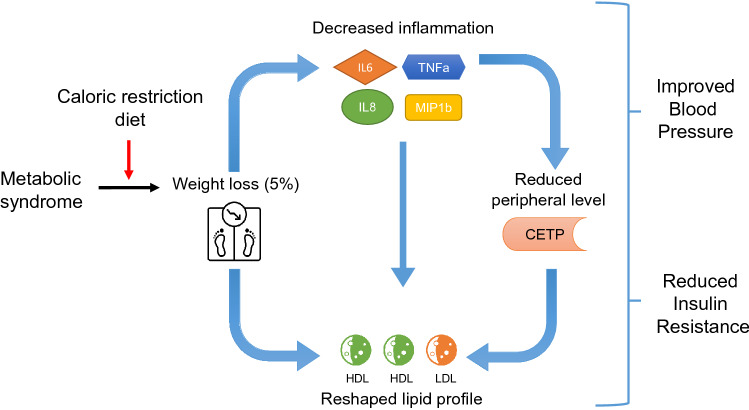

## Introduction

Prevalence of obesity and metabolic syndrome (MetS) are worldwide increasing to epidemic proportions, leading to increased risk of cardiovascular disease (CVD) and type 2 diabetes. The prevention of these diseases is a public health challenge. Diet, particularly Mediterranean diet, is one of the most important instruments to reduce obesity, MetS, cardiovascular diseases [1–3] and the risk of developing type 2 diabetes [4–7]. To this end, mechanisms whereby diet controls metabolic parameters are still poorly understood; however,, it seems that the pro-atherogenic inflammatory system may be involved [8]. It has been already demonstrated that diet may reduce circulating markers of inflammation in obese non-diabetic subjects [8, 9]. Moreover, this reduction seems to be directly linked to kind of food consumed (cereals, fruits, nuts, virgin oil) [5]. Conversely, some pro-inflammatory markers, such as IL-6 and C-reactive protein, may predict the risk of future type 2 diabetes and those are increased in MetS [9]. Interestingly, while robust association has been found among pro-inflammatory markers’ changes and glucose metabolism or insulin resistance changes, an association has not been equally demonstrated with lipid changes [8]. A major player in driving lipid changes is cholesteryl ester transfer protein (CETP), which enables the exchange of cholesteryl esters and triglycerides between high-density lipoprotein cholesterol (HDL) and triglyceride-rich lipoproteins. Reduced CETP activity leads to reduction of low-density lipoprotein cholesterol (LDL) and increased HDL [11] and consequently to significantly lower risk of atherosclerotic cardiovascular disease mainly in genetic studies [12], but not in pharmacological study aimed at targeting CETP activity [11]. Patients affected by type 2 diabetes showed increased CETP activity [13, 14]; whether CETP levels are associated with changes of lipid profile during weight loss has not been described yet. The aim of this study was to determine the effects of a diet-induced weight loss achieved with a balanced low-calorie diet on physical and biochemical metabolic parameters, on the pro-inflammatory profile and on the chemical composition of lipoproteins in patients suffering from overweight/mild obesity and metabolic syndrome.

## Subjects and methods

### Patients

**Fig. 1:**
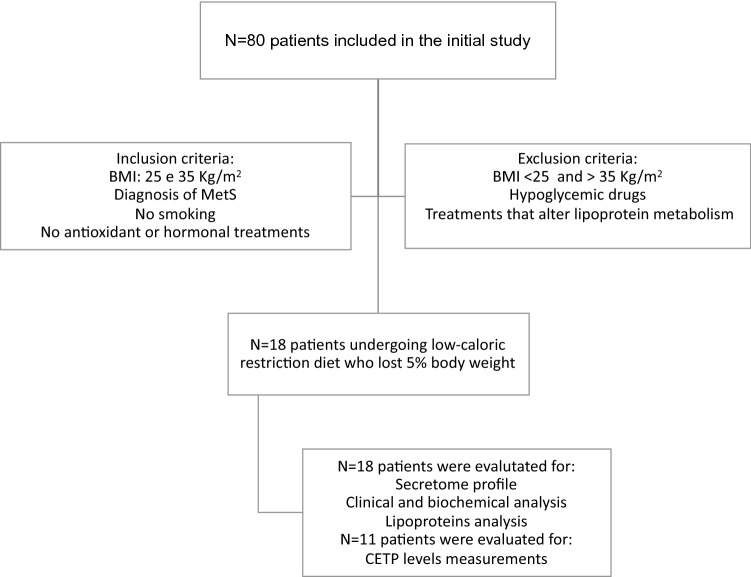
Flow-chart describing the study population

The subjects included in the present study were originally part of the “Oxidative Stress, Inflammation, and Lipoprotein in Metabolic Syndrome” study (ClinicalTrials.gov Identifier: NCT03553381). From this cohort we defined 18 overweight and moderately obese male Caucasian subjects with Body Mass Index (BMI) comprised between 25 and 35 kg/m^2^, who on enrolment had metabolic syndrome (MetS) defined according to International Diabetes Federation 2009 [15] and who after following a balanced hypo-caloric diet had lost at least 5% of their initial weight. Subjects were told and trained to reduce their daily energy intake of 800 kcal/day for 8 weeks with dietary counseling performed by a registered dietician. Macronutrient content of hypocaloric diet, expressed as percentage of ingested energy, was 25% fat, 60% carbohydrate and 15% protein. All subjects were non-smokers. Inclusion criteria also included the following: (i) alcohol consumption < 25 g/die, (ii) no use of antioxidant-based supplements and (iii) absence of hormonal treatments. Patients receiving hypoglycemic treatment, treatments that alter lipoprotein metabolism and pregnant women were also excluded (Fig. 1). The study was approved by the ethics committees of the Istituti Clinici di Perfezionamento of Milan and of L. Sacco Hospital of Milan and was carried out in accordance with the principles of the Declaration of Helsinki as revised in 2000. Subjects gave their written consent to the study. The primary endpoint of the study was to evaluate the change in the lipoprotein profile and the reduction in inflammation in patients who underwent a balanced hypo-caloric diet and lost at least 5% of their initial weight. Sample size was set at 18, as it would provide the study with 80% power to detect a reduction of at least 0.4 pg/ml in the cytokine levels after weight loss, with a significance level of *α* = 0.05, given that an increase of 0.4 pg/ml in IL-6 plasma level has been observed in patients with glucose intolerance [16].

### Blood collection and analyses

As previously described [17], overnight fast blood was drawn from subjects (12 h without food) in the morning at study entry and at the end of treatment after body weight loss. Blood collection and handling were carried out under strictly standardized conditions and in line with manufacturer recommendations. C-reactive protein level (CRP) was measured with the high-sensitivity assay. Plasma samples (EDTA as anticoagulant) were stored at − 80 °C until use. Lipoproteins were isolated from plasma as previously described [18] by adapting the procedure to “Optima Max” tabletop ultracentrifuge (Beckman Coulter). For a complete removal of albumin, the HDL fraction (density, 1.063–1.210 g/ml) was subjected to a second centrifugation [procedure 15 in ref. [18]]. After separation, lipoproteins were dialyzed and their levels of proteins, cholesterol (total and free), phospholipid and triacylglycerols were determined [18, 20].

### Lipopolysaccharide and lipopolysaccharide binding protein

Plasma lipopolysaccharide (LPS) levels were measured by Limulus amebocyte lysate test according to manufacturer instructions (Euroclone S.p.A, Milan, Italy). Plasma lipopolysaccharide binding protein (LBP) concentration was measured using an enzyme-linked immunosorbent assay kit (BioSource, Milan, Italy).

### Measurement of plasma cytokines

Levels of cytokines were assessed in plasma of patients enrolled in the study using the Bio-Plex Pro human cytokine 17-plex panel (M5000031YV, Bio-Rad) according to the manufacturer’s protocol [21]. The secretome profile was assessed at baseline (T0) and after diet/weight loss (T1) and a delta (T1–T0) of plasma cytokine level has been also calculated.

### Inflammatory score

Each plasma cytokine value was stratified into quartiles to determine cutoff points and assign a score ranging from 0, which was assigned to the lowest quartile, to 4, which was assigned to the highest quartile [22].

### Measurement of CETP

CETP levels were assessed using commercially available ELISA kit, according to the manufacturer’s instructions (MyBioSource, MBS266702, Milan, Italy). Human-derived hepatoma cell line (Huh7) was cultured for 5 days in Dulbecco’s Modified Eagle’s (DMEM) containing 10% Fetal Bovine Serum (FBS) at different glucose concentrations: 5.5, 20 and 35.5 mM as already described [23]. Culturing supernatant was collected, and CETP levels were assessed by ELISA.

### Statistical analysis

Data are expressed as median ± standard error of mean (SEM) unless otherwise reported. Since the Kolmogorov–Smirnov normality test revealed non-normal distribution, the results were analyzed by non-parametric tests. The effects of the hypo-caloric diet were analyzed by paired comparison (values before vs. after the intervention) using Wilcoxon tests. Two-tailed *p*-values ≤ 0.05 were considered significant. All statistical analyses were performed by using StatistiXL software (version 1.5; StatistiXL, Western Australia) and Prism Graphpad 7. A Pearson/Spearman correlation analysis was used as appropriate to define correlations between each cytokine level and CETP quantification, and also relations among improvement in glucose metabolism (glycemia reduction, insulin reduction, HOMA-IR reduction) and CEPT levels.

## Results

### Clinical characteristic at baseline and at the end of follow up

The anthropometric characteristics of patients, including blood pressure and blood parameter values before and after weight loss are summarized in Table 1. The mean age of the enrolled subjects was 47.5 ± 8.7 years and a 5% body weight loss was achieved in the study patients after a mean of 191.0 ± 46.2 days. All variables included in the metabolic syndrome improved significantly after diet and all patients reversed MetS. In particular, the diet treatment significantly improved BMI, waist circumference, systolic and diastolic blood pressure, fasting glycaemia, fasting insulin and HOMA-IR (homeostasis model assessment of insulin resistance). With regard to other biochemical parameters measured (serum protein, electrolyte, iron, uric acid, creatinine, thyroid hormone, white and red blood cells) no difference was observed, except for a slight reduction of CRP values although not reaching statistically significant difference (Table 1).

**Table 1:** Anthropometric characteristics, blood pressure and blood parameters of subjects before (T0) and after (T1) weight loss

	T0	T1
BMI (kg/m^2^)	34.7 ± 3.4	31.6 ± 2.9**
Waist circumference (cm)	113.0 ± 10.7	106.0 ± 8.4**
Systolic blood pressure (mmHg)	140.0 ± 15.5	128.0 ± 11.2**
Diastolic blood pressure (mmHg)	88.0 ± 9.4	79.0 ± 8.8**
Glycemia (mg/dL)	103.0 ± 22.5	97.0 ± 14.**
Insulin (µU/L)	16.1 ± 11.3	10.8 ± 5.4*
HOMA-IR	4.1 ± 3.0	2.5 ± 1.4**
HbA1c (mmol/mol)	5.6 ± 0.7	5.8 ± 0.2
Total cholesterol (mg/dL)	198.0 ± 30.6	198.0 ± 35.3
Triacylglycerols (mg/dL) LDL cholesterol (mg/dL) HDL cholesterol (mg/dL)	125.0 ± 66.4 123 ± 30.0 48 ± 12.8	135.0 ± 81.6 123 ± 29.6 48 ± 15.3
CRP (mg/L)	0.5 ± 0.35	0.4 ± 0.6

*BMI* body mass index, *HOMA-IR* homeostasis model assessment of insulin resistance, *CRP* C-reactive protein

*p ≤ 0.05

**p ≤ 0.01

### Lipid profile

While a change in total LDL and HDL levels was not evident, the analysis of extracted lipoproteins demonstrated that diet-induced weight loss also substantially reshaped lipoprotein chemical compositions. In fact, as shown in Table 2, a significant change in the concentration of triacylglycerols that increased in LDL while decreased in HDL (*p* < 0.01) was found. A significant increase in HDL Apo concentration has been observed after diet. Dietary treatment did not significantly influence LPS and LBP plasma levels (data not shown).

**Table 2:** Protein (Apo) and lipid concentrations of lipoproteins before (T0) and after (T1) weight loss

	VLDL		LDL		HDL	
	T0	T1	T0	T1	T0	T1
Apo (mg/dL)	9.8 ± 5.7	9.7 ± 5.4	12.3 ± 2.8	11.8 ± 2.4	9.0 ± 2.8	11.1 ± 2.8**
TC (mg/dL)	37.3 ± 20.7	34.4 ± 19.6	103.2 ± 29.9	108.6 ± 32.4	43.6 ± 11.7	42.8 ± 16.4
TAG (mg/dL)	60.0 ± 33.2	67.1 ± 42.5	45.3 ± 12.5	61.2 ± 16.2**	29.3 ± 7.8	21.9 ± 8.1**
PL (mg/dL)	18.7 ± 9.9	19.7 ± 11.6	72.2 ± 19.8	76.4 ± 19.7	47.8 ± 12.4	45.0 ± 14.2
TL (mg/dL)	116.3 ± 61.5	121.6 ± 76.1	220.7 ± 59.6	246.2 ± 66.7*	121.0 ± 31.5	110.3 ± 37.5*

*Apo* apolipoprotein; *TC* Total Cholesterol; *TAG* Triacylglycerols; *PL* Phospholipids; *TL* Total Lipids

*p ≤ 0.05

**p ≤ 0.01

### Peripheral pro-inflammatory cytokines profile

**Fig. 2:**
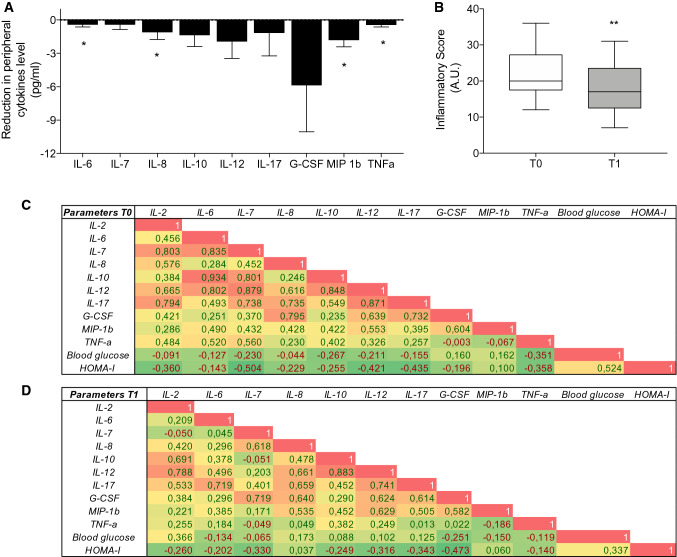
**a** Relative variation in peripheral cytokine levels of patients included in the study. Cytokines levels are expressed in pg/mL. **b** Inflammatory score assessed in patients before (T0) and after weight loss (T1). **c, d** Correlation matrix between cytokines level and clinical parameters before (T0) and after weight loss (T1). *IL* interleukin; *TNF* tumor necrosis factor; *G-CSF* Granulocyte-Colony Stimulating Factor; *MCP1* Monocyte Chemoattractant Protein 1; *MIP1b* Macrophage Inflammatory Protein 1b; *A.U.* Arbitrary Unit; *HOMA-IR* homeostasis model assessment of insulin resistance. *p ≤ 0.05; **p < 0.01

**Fig. 3:**
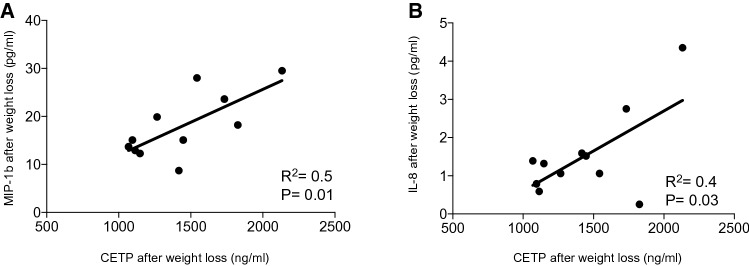
Correlation between MIP-1b (**a**), IL8 (**b**) and IL10 (**c**) with CETP level after weight loss in the 18 patients. **a** Correlation between MIP1b (Macrophage Inflammatory Protein 1b expressed in pg/mL) and CETP (cholesterol ester transfer protein expressed in ng/mL) after weight loss. **b** Correlation between IL-8 (Interleukin 8 expressed in pg/mL) and CETP (cholesterol ester transfer protein expressed in ng/mL) after weight loss

The quantification of cytokines is reported in Table 3, with data showing a significant decrease of the peripheral levels of IL-6, TNF-α, IL-8 and MIP-1β (Table 3). Moreover, for other cytokines, such as IL-17, GM-CSF and MCP-1, a trend toward a downregulation, albeit non statistically significant, was observed (Fig. 2a). We next stratified cytokines levels based on quartiles and calculated an inflammatory score, which summarizes the overall inflammatory state detected in the periphery. Our results further confirmed that a higher inflammatory state was evident in patients before weight loss and that dietary treatment was able to reshape it (Fig. 2c, d). Finally, unchanged CETP peripheral blood levels have been observed after weight loss (Table 3). However, an interesting correlation between CETP levels and different peripheral cytokine levels, in particular MIP-1b and IL-8, has been observed after weight loss (Fig. 3a, b), while that between reduction of cytokines levels and CETP did not result statistically significantly different. Spearman’s correlation analysis was also performed among CEPT level and glycemia levels, insulin levels and HOMA-IR index, but no significant correlations have been found (data not shown). In order to understand the basis for influence of cytokine changes on CEPT production, which is primarily released by the liver, we cultured a human hepatocytes-derived cell line (Huh7) in vitro with sera obtained from patients with MetS before and after diet and did not demonstrate a significant change in supernatant secretion of CETP. Similarly, no differences have been observed among supernatant CETP levels from human hepatocyte-derived cell line (Huh7) cultured in vitro with sera obtained from patients with MetS as compared to those obtained from healthy controls (data not shown).

**Table 3:** Peripheral cytokine and CETP levels before (T0) and after (T1) weight loss

	T0	T1
IL-6 (pg/mL)	1.9 ± 0.7	1.5 ± 0.6*
TNFα (pg/mL)	0.8 ± 0.3	0.3 ± 0.8*
IL-8 (pg/mL)	2.7 ± 0.6	1.6 ± 0.3*
IL-10 (pg/mL)	3.5 ± 1.2	2.5 ± 0.6
IL-12 (pg/mL)	8.1 ± 2.2	6.2 ± 1.8
IL-7 (pg/mL)	1.3 ± 0.4	1.0 ± 0.2
IL-17 (pg/mL)	8.0 ± 2.7	6.9 ± 2.4
G-CSF (pg/mL)	17.9 ± 3.8	12.1 ± 2.6
MIP-1β (pg/mL)	20.2 ± 1.4	18.4 ± 1.4*
MCP-1 (pg/mL)	31.4 ± 4.5	31.6 ± 4.5
CETP (ng/mL)	356.2 ± 57.4	405.8 ± 97.9

Data are expressed as mean ± SEM (standard error of the mean)

*IL-2* interleukine 2; *IL-6* interleukine 6; *IL-7* interleukine 7; *IL-8* interleukine 8; *IL-10* interleukine 10; *IL-12* interleukine 12; *IL-17* interleukine 17; *GM-CSF* Granulocye-Macrophage Colony Stimulating Factor; *MCP1* Monocyte Chemoattractant Protein 1; *MIP1b* Macrophage Inflammatory Protein 1b.; *CETP* cholesterol ester transferase protein

*p ≤ 0.05

## Discussion

Our study showed the efficacy of body weight loss obtained through caloric restriction in reducing inflammatory cytokine levels and in reshaping lipid composition. In previous studies adherence to Mediterranean diet led to an improvement in clinical cardiovascular outcomes with a reduction 10–30% in relative risk of CVD [6], to a protection against recurrent coronary heart disease [3], to a reduction of 18–40% in incident diabetes and to a reduction of 14% in the prevalence of MetS [6, 7]. The results of present study show as expected [1, 5], an efficacy of balanced caloric restrictions in reverting MetS by reducing body weight, associated with a reduction in blood pressure, glycaemia levels and insulin resistance. Interestingly in this cohort, balanced caloric restriction leads also to an improvement in lipid composition, with an increase in the triacylglycerol concentration in LDL and with a decrease in HDL, and an increase in Apo concentration in HDL. It is also known that obesity is associated with a chronic low-grade inflammatory state of adipose tissue and that this independently increases the risk of adverse cardiovascular outcomes [24, 25]. Previous diet intervention studies showed that weight loss has positive effect on pro-inflammatory cytokines levels, particularly it is effective in reducing IL-6 and CRP levels, but this cytokine reduction was not related to significant lipids levels and composition improvement [8, 26]. Interestingly, in our study we described a significant reduction of not only several inflammatory cytokines such as IL-6 but also IL-8, TNF-α and MIP-1b after diet, which was ultimately summarized in a reduction of the inflammatory score upon dietary treatment. The aforementioned cytokines have been mainly linked to the innate immunity response [27] and we may suggest that the metabolic syndrome itself may promote an increased release of cytokines by circulating monocytes. Given the beneficial effect obtained in reducing those cytokines’ level through the weight loss in our study, in which a reduction of the waist was evident, we may further speculate that adipocytes residing in the visceral adipose tissue may also be responsible for the cytokine production. Moreover, we have found a correlation between the levels of different inflammatory cytokines and CETP levels after diet, in particular lower IL-8 and MIP-1b levels were associated with lower CETP level, which has been associated with the formation of HDL and a reduced the risk of atherosclerosis [28]. Beyond the role in the lipid exchanges among lipoprotein classes, CETP has also postulated roles in inflammatory processes and in the immunological defenses of the organism [29]. This has been also suggested by our study, which showed a link between peripheral CETP level and that of some pro-inflammatory cytokines, such as IL-8 and MIP-1b, with an emerging key-role in the field of metabolic disease and diabetes. Further studies are needed to better unveil the relationship between lipid changes, pro-inflammatory cytokine profile modifications and CEPT, both level and activity. Finally, we acknowledge that the lack of a control group and the small sample size are probably a limitation of this study and further larger case–control studies would be required to confirm our results. However, our study demonstrates that a link exists between lipids and pro-atherogenic inflammatory cytokines and that it can be strongly modulated by diet. In conclusion, body weight loss through balanced caloric restriction in patients with metabolic syndrome leads to a protective anti-atherogenic lipid profile and a reduced peripheral inflammatory environment, which are both associated with a decrease in cardiovascular risk.
